# Probiotic *Escherichia coli* inhibits biofilm formation of pathogenic *E. coli* via extracellular activity of DegP

**DOI:** 10.1038/s41598-018-23180-1

**Published:** 2018-03-21

**Authors:** Kuili Fang, Xing Jin, Seok Hoon Hong

**Affiliations:** Department of Chemical and Biological Engineering, Illinois Institute of Technology, Chicago, IL 60616 USA

## Abstract

Many chronic infections involve bacterial biofilms, which are difficult to eliminate using conventional antibiotic treatments. Biofilm formation is a result of dynamic intra- or inter-species interactions. However, the nature of molecular interactions between bacteria in multi-species biofilms are not well understood compared to those in single-species biofilms. This study investigated the ability of probiotic *Escherichia coli* Nissle 1917 (EcN) to outcompete the biofilm formation of pathogens including enterohemorrhagic *E. coli* (EHEC), *Pseudomonas aeruginosa*, *Staphylococcus aureus*, and *S. epidermidis*. When dual-species biofilms were formed, EcN inhibited the EHEC biofilm population by 14-fold compared to EHEC single-species biofilms. This figure was 1,100-fold for *S. aureus* and 8,300-fold for *S. epidermidis*; however, EcN did not inhibit *P. aeruginosa* biofilms. In contrast, commensal *E. coli* did not exhibit any inhibitory effect toward other bacterial biofilms. We identified that EcN secretes DegP, a bifunctional (protease and chaperone) periplasmic protein, outside the cells and controls other biofilms. Although three *E. coli* strains tested in this study expressed *degP*, only the EcN strain secreted DegP outside the cells. The deletion of *degP* disabled the activity of EcN in inhibiting EHEC biofilms, and purified DegP directly repressed EHEC biofilm formation. Hence, probiotic *E. coli* outcompetes pathogenic biofilms via extracellular DegP activity during dual-species biofilm formation.

## Introduction

Biofilms are sessile microbial communities that form on biotic and abiotic surfaces through the secretion of extracellular polymeric substances^1^ that enhance adherence to the surfaces and microbial aggregation^2^. Biofilm bacteria are highly tolerant to exogenous stresses, such as antibacterial agents, due to mass transfer limitations in the biofilm matrix as well as the non-metabolizing nature of cells inside the biofilms^3^. Numerous chronic and medical device-related infections are caused by bacterial pathogens living in biofilm communities due to these biofilm properties^1,4^. Therefore, understanding biofilm formation is critical to establishing novel strategies for controlling infectious diseases.

Although the majority of biofilm studies have examined single-species cultures^5^, several notable studies have investigated mixed species interactions. Indole production by *E. coli* promotes its growth and biofilms but represses the quorum sensing signals of *P. aeruginosa*^6^ as well as other Gram-negative bacteria^7^. In contrast, iron sequestration or phenazine production by *P. aeruginosa* represses *E. coli* growth^8^. While planktonic cells are susceptible to environmental cues, mixed-species biofilm communities provide a stable environment, *e.g*., they maintain viable phage and bacterial populations^9^ or induce adaptive bacterial physiological responses^10^. Molecular interactions between pathogenic and non-pathogenic bacteria are also potentially important in understanding bacterial colonization patterns, as shown in the bacterial community parameters in central venous catheters^11^.

Biofilm formation is an intra- and inter-species phenomenon that requires dynamic interactions between bacteria in mixed biofilm communities^12^. The interactions are cooperative between biofilm bacterial species via cell-cell communication, metabolic cooperation, or spatial organization^5^. For example, aerobic and anaerobic microorganisms coexist by mediating redox metabolism, such as sulfide oxidation, to enhance mutual cell growth in biofilms^13^. *Enterococcus faecalis* modulates the local environment to promote the growth and biofilm formation of *E. coli*, a co-infecting organism of urinary tract infections^14^. Notably, biofilm growth is also governed by competitive interactions to exclusively uptake nutrients or occupy spatial resources^12^. Some microorganisms outcompete other species by producing anti-biofilm agents such as non-biocidal biosurfactants, enzymes, and metabolites that inhibit all levels of biofilm development, disrupt cell-cell communication, or enhance the dispersal of target biofilms^15^. In addition, consortial biofilms can be controlled by engineering cellular signaling. For instance, one biofilm inhibits or eradicates another biofilm by utilizing quorum sensing signals known as autoinducers and biofilm dispersal proteins^16^, interspecies-biofilm signal indole^17^, and the antimicrobial peptides indolicidin and bactenecin^18^. Hence, both cooperative and competitive behaviors between bacterial species determine biofilm formation dynamics in mixed bacterial communities.

Probiotics are live microorganisms that are beneficial to the host^19^. Probiotics adhered to the intestinal epithelial tissue enhance epithelial barrier function, which is essential to the host defense system in preventing infection and inflammation from pathogens^20^. *Lactobacillus* species, *Bifidobacterium* species, *Escherichia coli*, and *Streptococcus* species commonly enhance the host immune system in actions that occur either individually or in combination^20^. Probiotic *E. coli* Nissle 1917 (EcN) has been applied for human and veterinary clinical treatments to mitigate intestinal disorders such as ulcerative colitis and inflammatory bowel disease^21–23^. When EcN colonizes on the surface of the intestine together with many intestinal microorganisms including probiotic, non-probiotic, and pathogenic bacteria, EcN stimulates intestinal epithelial cells to produce human β-defensin 2. This improves host immune response^24^, promotes the secretion of microcins as strain-specific antimicrobials^25^, and inhibits the growth and biofilm formation of the other *E. coli* strains^26,27^. However, how EcN outcompetes the biofilm formation process of other *E. coli* strains and whether EcN has the ability to inhibit biofilms of bacterial species other than the *E. coli* strains remain poorly understood.

This study investigates molecular interactions between EcN and pathogens when they form dual-species biofilms and elucidates the related inhibition mechanisms by identifying relevant protein molecules. We chose four pathogens to assess the population dynamics with EcN in dual-species biofilms. These pathogens include enterohemorrhagic *E. coli* O157:H7, which causes diarrhea or hemorrhagic colitis in humans^28^, *Pseudomonas aeruginosa*, which is the primary cause of lung infection resulting in cystic fibrosis^29^, and *Staphylococcus aureus* and *S. epidermidis*, which are two of the most important pathogens contaminating medical implants and instruments^30,31^. We observed that EcN inhibits the biofilm formation of *E. coli* strains, *S. aureus*, and *S. epidermidis* but not of *P. aeruginosa*. We performed mass spectrometry of *E. coli* supernatants, assessed the biofilm formation of pathogens with an EcN mutant lacking *degP*, and investigated the addition of purified DegP during biofilm formation. We identified that extracellular DegP secreted from EcN represses other biofilms.

## Results

### EcN inhibits the biofilm formation of other *E. coli* strains

**Figure 1:**
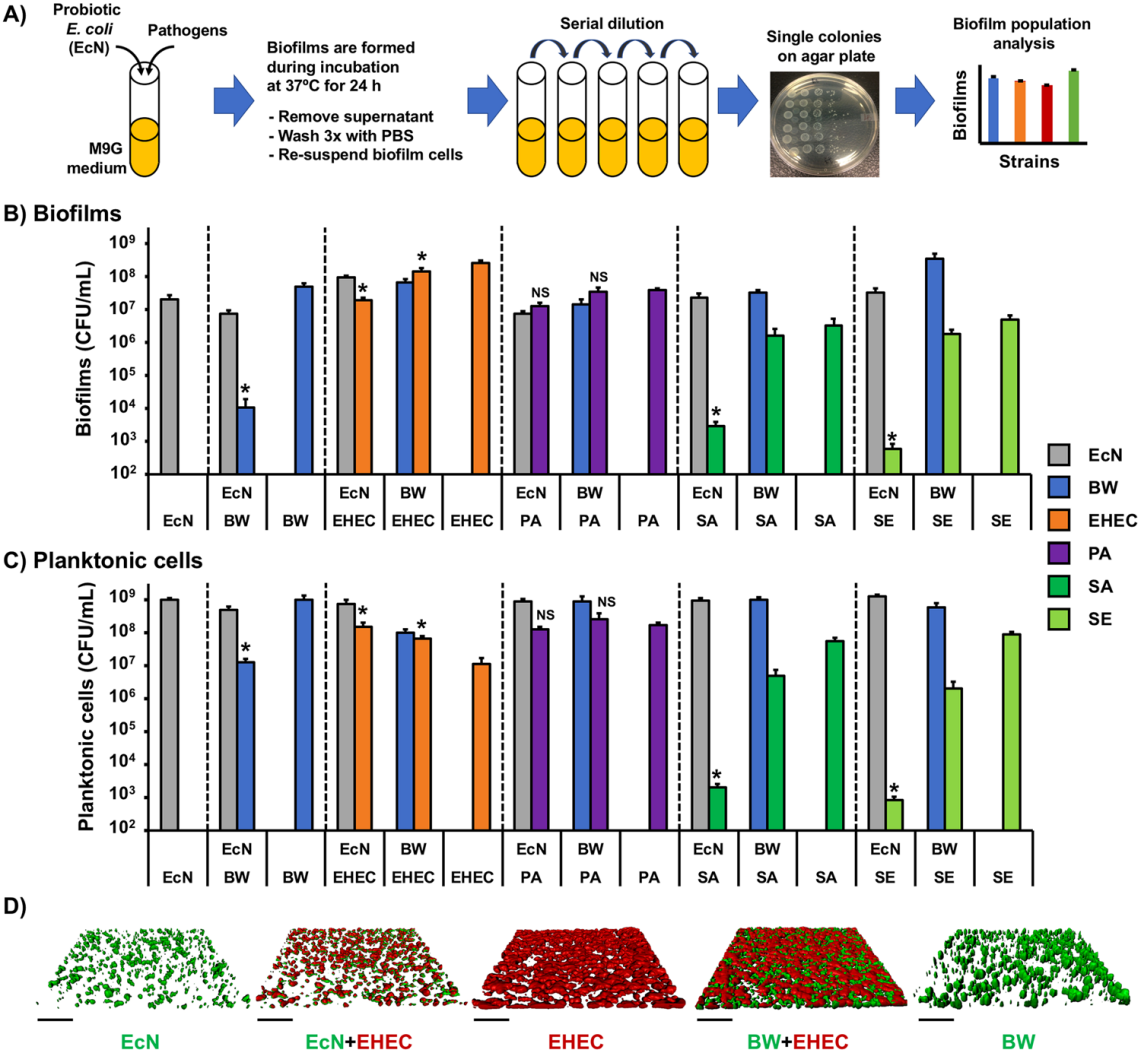
Single- and dual-species biofilm formation of probiotic *E. coli* and pathogens. (**A**) Scheme of dual-species biofilm quantification. (**B**) Single- and dual-species biofilms were formed in polypropylene culture tubes in M9G for 24 h at 37 °C without shaking, and viable biofilm cells were quantified by colony forming units (CFU). Probiotics *E. coli* Nissle 1917 (EcN), commensal *E. coli* BW25113 (BW), enterohemorrhagic *E. coli* EDL933 (EHEC), *P. aeruginosa* PAO1 (PA), *S. aureus* JE2 (SA), and *S. epidermidis* RP62A (SE) were tested to determine the effect of EcN towards biofilm formation of the other bacteria. (**C**) Planktonic cell density at the same condition with (**B**) was quantified. Significant differences of pathogenic biofilms in dual-species biofilms (or planktonic cells) to its single-species biofilms (or planktonic cells) were analyzed by a two-tailed *t*-test for (**B**) and (**C**). Each data point is the average of at least six independent cultures, and one standard deviation is shown. * represents a significant difference with a p-value < 0.01. NS indicates ‘not significant’. (**D**) Single- and dual-species biofilm images of EcN, BW, and EHEC using a confocal microscope. Biofilms were formed in 24-well plates in M9G medium at 37 °C for 24 h. Scale bar represents 100 μm.

EcN represses the biofilms of intestinal *E. coli* strains during dual-species biofilm formation^26^ and inhibits the cell growth of *Salmonella enterica*^25,32^; we thus hypothesized that EcN may repress the biofilm formation of other pathogens. To determine the probiotic effects of EcN, we investigated the single- and dual-species biofilm formation of pathogens with EcN. The strains tested in this study were commensal *E. coli* BW25113^33^ as a control, enterohemorrhagic *E. coli* EDL933^28^, *P. aeruginosa* PAO1^34^, *S. aureus* JE2^35^, and *S. epidermidis* RP62A^36^. These strain names were abbreviated to BW, EHEC, PA, SA, and SE, respectively. We chose a minimal medium with 0.4% glucose (M9G) to determine sufficient biofilm formation and inhibition in our biofilm study because a limited nutrient supply resulted in the formation of robust EHEC biofilms compared to that in a rich medium (Supplementary Fig. 1). To distinguish each strain population in the dual-species biofilms, we used the strains exhibiting resistances to different antibiotics (Supplementary Table 1). Dual-species biofilms were formed in M9G medium without antibiotics. The isolated biofilm cells were spread on two types of LB agar plates, each containing different antibiotics. Only the cells expressing the corresponding antibiotic resistance gene grew on the agar plate during overnight incubation and were regarded as one population of dual-species biofilms. For EcN and BW strains, we also used wild-type strains with no antibiotic resistance depending on the antibiotic resistance of the other strain and were able to distinguish two populations by subtracting the antibiotic-resistant biofilm population from the count of the total dual-species biofilms. We confirmed that wild-type and antibiotic-resistant EcN or BW formed similar levels of biofilms (Supplementary Fig. 2). We seeded the same concentrations (approximately 4 × 10^6^ CFU/mL) of EcN and other strains (Fig. 1A) in M9G medium and incubated them at 37 °C for 24 h to form the single- or dual-species biofilms. After 24 h of incubation, the level of EcN biofilms in the single-species culture was 2.1 ± 0.7×10^7^ CFU/mL (Fig. 1B). EcN inhibited the EHEC biofilm population by 14-fold in the dual biofilms compared to the EHEC single biofilms while also increasing the EcN biofilm population by 8-fold in the dual biofilms (Fig. 1B). Interestingly, the EHEC single biofilms were 12-fold more populous than the EcN single biofilms (Fig. 1B), while the EcN planktonic culture was 90-fold greater than the EHEC planktonic culture **(**Fig. 1C), implying that EHEC adhesion to the surface or between EHEC cells may be more efficient than EcN adhesion. Thus, when dual biofilms are formed between EcN and EHEC, EHEC biofilm cells may work as adhesion points for promoting the initial attachment and biofilm growth of EcN prior to inhibiting EHEC biofilms. EcN also decreased non-probiotic and non-pathogenic *E. coli* BW biofilm formation by 4,700-fold compared to BW single biofilms (Fig. 1B). It is possible that EcN acidifies the culture media during dual biofilm formation. However, we observed that the pH of the EcN supernatant (6.26 ± 0.07) from the 24-h incubated culture was between the pH of the EHEC supernatant (5.9 ± 0.4) and BW supernatant (6.7 ± 0.1), indicating that EHEC and BW biofilm inhibition by EcN are not due to acidification induced by the culture. In addition, the reduction in EHEC biofilms due to EcN was not caused by a decrease in cell growth because EcN did not inhibit the growth of EHEC but rather increased the planktonic EHEC density by over 13-fold in the dual culture compared to the EHEC single culture **(**Fig. 1C). Additionally, there was no correlation between cell growth and biofilm formation in the *E. coli* strains^26^. Similarly, confocal microscopy observation and an analysis of single- and dual-species biofilms showed a 12-fold biomass reduction in EHEC by EcN but nearly no reduction by BW (Fig. 1D and Supplementary Table 2). These results corroborate the report that EcN represses the biofilms of intestinal *E. coli* strains during dual-species biofilm formation^26^ and confirm the probiotic effects of EcN toward other *E. coli* strains.

### EcN inhibits biofilm formation of *S. aureus* and *S. epidermidis*

Next, we investigated whether EcN can influence other pathogens during dual-species biofilm formation. We did not observe a decrease in PA biofilms when co-cultured with EcN or BW (Fig. 1B), implying that the biofilm formation of PA is insensitive to the presence of *E. coli* strains. The skin pathogens SA and SE showed substantial decreases in their biofilm formation by 1,100- and 8,300-fold, respectively, in the dual-species biofilms formed with EcN compared to their single-species biofilms (Fig. 1B). However, when testing the effect of non-probiotic *E. coli* with SA and SE, we did not observe a decrease in their biofilm formation. In addition, we found that the growth rates of SA and SE in dual culture with EcN were decreased by approximately 3-fold compared to those in the single culture (Supplementary Table 3) and that the planktonic cell densities of SA and SE after 24 h growth in dual culture with EcN were significantly lower (by four orders of magnitude) than those of the single culture (Fig. 1C). These results suggest that the probiotic strain EcN may inhibit the growth of SA and SE, contributing to their biofilm reduction.

### EcN supernatant inhibits biofilm formation of EHEC

**Figure 2:**
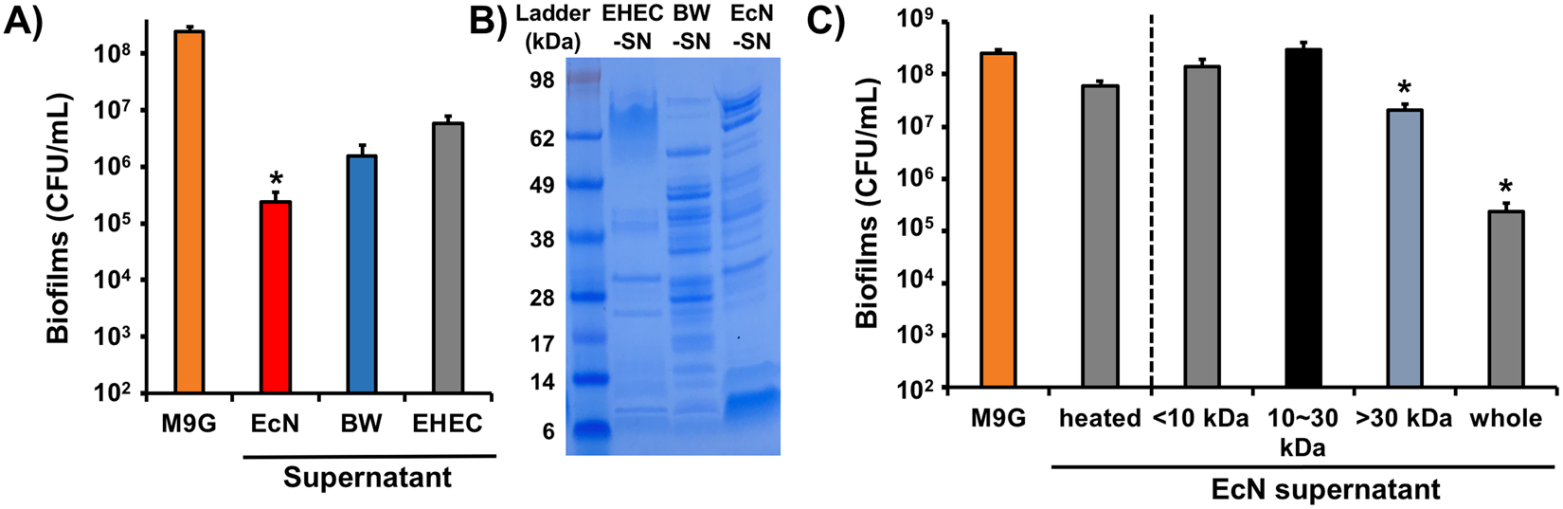
Effect of cell-free supernatants in EHEC biofilm formation. (**A**) EHEC biofilm formation in EcN, BW, and EHEC supernatants at 37 °C for 24 h. Each data point is the average of at least six independent cultures, and one standard deviation is shown. * represents a significant difference with a p-value < 0.01 by a two-tailed *t*-test. (**B**) Protein profiles of EcN, BW, and EHEC supernatants on SDS-PAGE gel. Full SDS-PAGE gel is shown in Supplementary Fig. 8A. (**C**) EHEC biofilm formation in heated EcN supernatant and EcN supernatants with different molecular weights at 37 °C for 24 h. Each data point is the average of at least two independent cultures with triplicates, and one standard deviation is shown. * represents a significant difference with a p-value < 0.01 by a two-tailed *t*-test.

Because EcN inhibited the biofilms of EHEC, SA, and SE when co-cultured whereas BW did not (Fig. 1B), we reasoned that EcN may produce and release molecules that repress other biofilms. We prepared cell-free supernatants of the overnight cultures of EcN, BW, and EHEC grown in M9G medium by removing all cells in the culture via centrifugation and filtration. We then investigated EHEC biofilm formation in the supernatant media to determine if EHEC biofilm formation is repressed in the EcN supernatant. We observed that the EcN supernatant decreased EHEC biofilm formation up to 1,000-fold, whereas the BW and EHEC supernatants decreased EHEC biofilm formation by approximately 40- to 160-fold (Fig. 2A). The nutrient composition of the supernatants prepared from the overnight cultures may substantially differ from the fresh M9G medium because bacteria secrete many proteins (Fig. 2B) and metabolites during their growth; differences may also be due to bacterial cell lysis, which may have influenced EHEC biofilm formation in the BW and EHEC supernatants. However, if the EcN supernatant contained the biofilm-inhibiting molecules secreted from EcN, the effect of EHEC biofilm reduction by the EcN supernatant would be more significant than that of the other supernatants. Indeed, we observed a 1,000-fold biofilm reduction of EHEC in the EcN supernatant (Fig. 2A). The net contribution of the EcN supernatant toward EHEC biofilm inhibition was approximately 10-fold, as demonstrated by eliminating the contribution of non-probiotic BW and EHEC supernatants by division; this was consistent with the EHEC biofilm inhibition by EcN in dual biofilms (Fig. 1B). Thus, these results imply that probiotic *E. coli* secretes molecules that effectively inhibit EHEC biofilm formation.

### Microcins are not responsible for biofilm inhibition of other strains

The dual-species biofilm results (Fig. 1B) and EHEC biofilms in the EcN supernatant (Fig. 2A) clearly indicate that the molecules secreted by EcN are potentially toxic to the other cells. EcN is known to suppress enterobacteria that utilize catecholate siderophores because the microcins M and H47 secreted from EcN can bind to the siderophore receptors and inhibit iron uptake to the cells^25^. A recent study showed that the production and secretion of microcins in EcN limit the expansion of competing *Enterobacteriaceae* during intestinal inflammation^32^. However, the ability of microcins to inhibit other biofilms was not clearly understood^26^. To determine whether microcins secreted from EcN function as inhibitors of other strains tested in our dual-species biofilms, we compared wild-type EcN to the mutant strain lacking microcins (EcNΔm) for the dual-species biofilm formation with EHEC. To completely remove microcin production, we knocked out genes encoding microcin M and H47 on the EcN chromosome (Supplementary Fig. 3A) via a one-step inactivation method using λ Red recombinase^37^. The biofilm formation of EcNΔm was similar to that of the EcN wild-type in single-species biofilms (Supplementary Fig. 3B), and the level of EHEC biofilm reduction by EcNΔm was similar to that by EcN wild-type (Supplementary Fig. 3C). Therefore, microcins are not responsible for inhibiting EHEC biofilms.

### High-molecular-weight proteins from probiotic *E. coli* inhibit EHEC biofilms

To identify the candidate molecules from EcN inhibiting the biofilms of EHEC, we first compared the EHEC biofilm formation in EcN supernatant with or without heat treatment. We incubated the EcN supernatant at 70 °C for 10 min to denature the proteins contained in the supernatant. In the heated EcN supernatant, EHEC biofilms were barely decreased (Fig. 2C), whereas the EHEC biofilms in the non-heated supernatant were decreased by approximately 1,000-fold, implying that proteins rather than small metabolites are responsible for inhibiting EHEC biofilms. Next, we separated the EcN supernatant into three molecular weight (MW) classes: MW > 30 kDa, 10 kDa < MW < 30 kDa, and MW < 10 kDa, and tested EHEC biofilm formation in these supernatants. EHEC biofilms were reduced by more than 10-fold when the MW of the components in the supernatant was greater than 30 kDa, while there was no inhibition observed in the lower MW supernatants (Fig. 2C). Furthermore, these results confirm that microcins do not affect EHEC biofilm formation (Supplementary Fig. 3C), as the molecular weights of microcins M and H47 are 9.0 kDa and 6.6 kDa, respectively. Thus, we concluded that the molecules secreted from EcN that inhibit EHEC biofilms are possibly proteins greater than 30 kDa.

### Mass spectrometry analysis of EcN supernatant reveals proteins secreted from EcN

**Figure 3:**
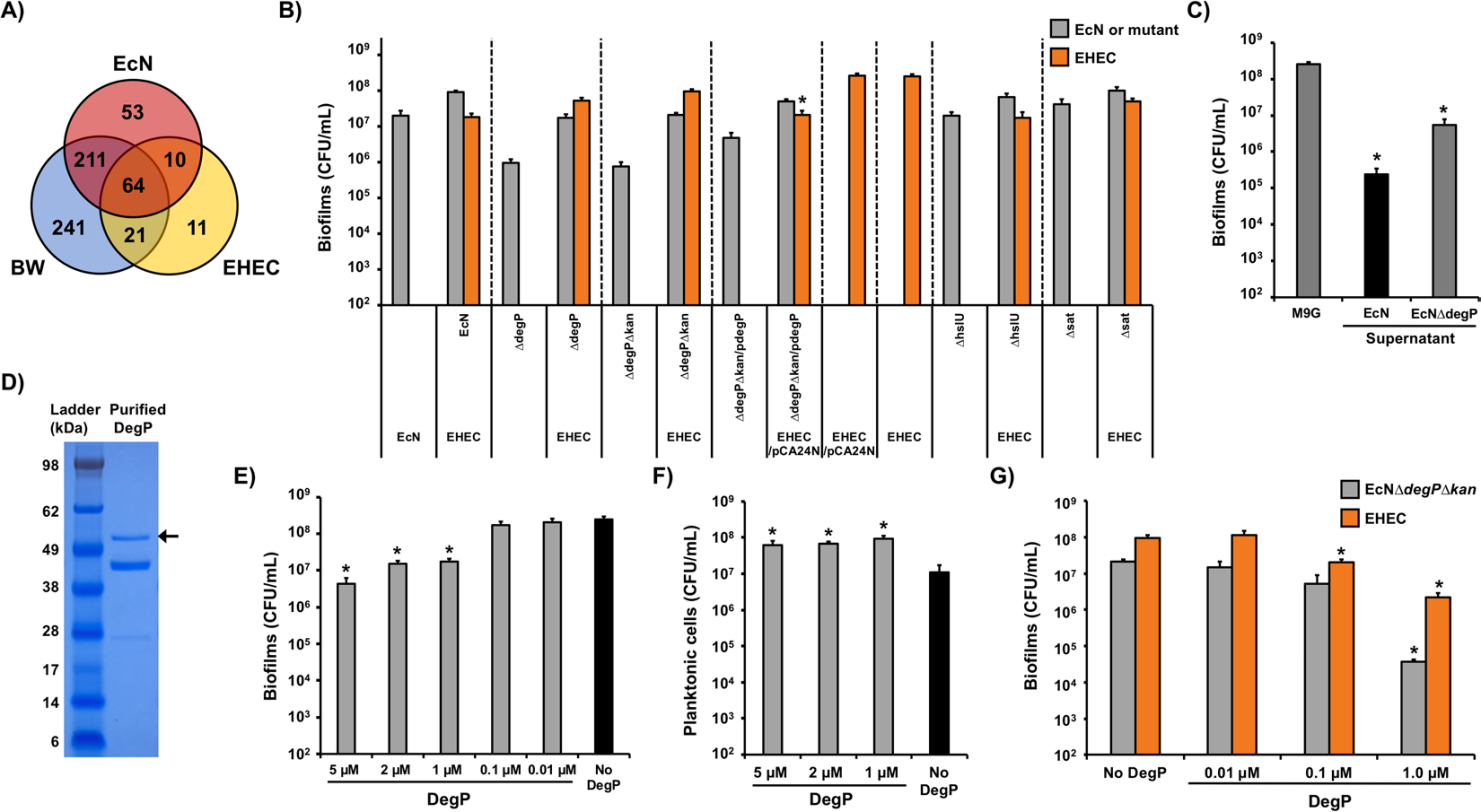
Analysis of proteins secreted from EcN and effect of DegP on EHEC biofilms. (**A**) The number of proteins in the EcN, BW, and EHEC supernatants identified from mass spectrometry analysis. (**B**) Single-species biofilms of EcN mutants (EcN∆*degP* (∆degP), EcN∆*degP*∆*kan* (∆degP∆kan), EcN∆*degP*∆*kan*/pCA24N-*degP* (∆degP∆kan/PdegP), EcN∆*hslU* (∆hslU), and EcN∆*sat* (∆sat)) and dual-species biofilms between EcN mutants and EHEC were formed in M9G for 24 h at 37 °C without shaking. (**C**) EHEC biofilm formation in supernatants from EcN wild-type and EcN∆*degP* at 37 °C for 24 h. (**D**) DegP protein purified through Strep-tag was shown on the SDS-PAGE gel. DegP with Strep-tag is 50.4 kDa, indicated with an arrow. Full SDS-PAGE gel is shown in Supplementary Fig. 8B. EHEC biofilms (**E**) and planktonic cells (**F**) with different concentrations of DegP at 37 °C for 24 h. (**G**) Dual biofilms between EcN∆*degP*∆*kan* and EHEC with different concentrations of DegP at 37 °C for 24 h. For (**B**), (**C**), (**E**), (**F**), and (**G**), each data point is the average of at least two independent cultures with triplicates, and one standard deviation is shown. * represents a significant difference with a p-value < 0.01 by a two-tailed *t*-test.

During EcN growth, varieties of proteins were secreted to the outside of the EcN cells, and some of these proteins were distinct from the proteins in the BW and EHEC supernatants (Fig. 2B). To identify the secreted proteins from EcN, we performed tandem mass spectrometry analysis and compared the protein profiles of the EcN supernatant to those of the BW and EHEC supernatants. A total of 338 proteins were detected in the EcN supernatant, 537 proteins in the BW supernatant, and 106 proteins in the EHEC supernatant (Fig. 3A). The supernatants may have contained the secreted proteins from active protein secretion pathways as well as the internal cellular proteins resulting from cell lysis during overnight growth. We analyzed 53 proteins that were present only in the EcN supernatant to identify possible candidates in inhibiting EHEC biofilms (Supplementary Table 4). Although the majority of proteins in the EcN supernatant were related to the flagella, cellular metabolism, and transcription/translation, three proteins (DegP, HslU, and Sat) were identified with a function of protease activity with protein sizes of greater than 30 kDa.

### Deletion of *degP* in EcN abolishes the inhibition of EHEC biofilms

We previously reported that proteases are important in mediating biofilm dispersal^38^. We hypothesized that the secretion of proteins with protease activity from EcN may lead to the inhibition of EHEC biofilm formation. DegP is a chaperone as well as a protease that degrades aggregated or denatured proteins from the inner-membrane and periplasmic space in *E. coil*^39^. HslU is a component of the ATP-dependent protease complex HslUV^40^ that enhances the peptide hydrolysis of HslV via the ATPase activity of HslU^41^. Sat is a secreted autotransporter toxin and, upon secretion from uropathogenic *E. coli*, stimulates cytoplasmic vacuolation of bladder and kidney epithelial cells^42^. Hence, the activities of DegP, HslU, and Sat may be related to the functions of EcN in inhibiting other pathogenic bacteria during biofilm formation. To reveal the contribution of these proteins in inhibiting EHEC biofilms, we knocked out *degP* (*ECOLIN_00860*), *hslU*, and *sat* (*ECOLIN_16105*) genes individually in the EcN wild-type strain and tested the single- and dual-species biofilms between each EcN mutant and EHEC. The deletion of *hslU* or *sat* in EcN did not alter their single-species biofilm populations and still exhibited EHEC biofilm repression to an extent that was similar to that of the EcN wild-type (Fig. 3B). In contrast, the single-species biofilm formation of the EcN strain lacking *degP* expression (∆*degP*) was 20-fold lower than that of the EcN wild-type, and EcN∆*degP* lost the ability to inhibit EHEC biofilms in dual-species biofilms (Fig. 3B). To determine if there was a polar effect upon the deletion of *degP*, we removed the kanamycin resistance gene cassette from the *degP* mutant. Similar to EcN∆*degP*, EcN∆*degP*∆*kan* did not exhibit EHEC biofilm inhibition (Fig. 3B). Furthermore, the expression of *degP* with a leaky promoter of pCA24N-*degP* without induction^43^ in the *degP*-deleted background (∆*degP*∆*kan*/P*degP*) restored EcN wild-type behavior in inhibiting EHEC biofilms (Fig. 3B); however, we did not observe the EHEC biofilm inhibition when *degP* was overexpressed (Supplementary Fig. 4A) due to its toxicity to the host cells upon induction (Supplementary Fig. 4B).

It is possible that the reduced EHEC biofilm inhibition by the *degP*-deleted EcN came from the low biofilm formation of the *degP*-deleted EcN. To demonstrate that the reduced EHEC biofilm inhibition by EcN∆*degP* was not from a low level of biofilm formation of EcN∆*degP* but instead from the absence of an interaction through DegP protein, we examined EHEC biofilm formation in the cell-free supernatant prepared from the culture of EcN∆*degP*. In the absence of DegP in the supernatant medium, the level of EHEC biofilms was not decreased to as low as that in the supernatant from the EcN wild-type (Fig. 3C) but was similar to the case with the supernatant from commensal *E. coli* BW (Fig. 2A), which did not show any inhibition effect toward other biofilms (Fig. 1B). Therefore, DegP production may be necessary for EcN to inhibit EHEC biofilms.

### DegP represses EHEC biofilms

To further support the extracellular function of DegP in inhibiting EHEC biofilms, we investigated the effect of purified DegP during EHEC biofilm formation. We first confirmed the purified DegP, showing an expected protein size (50.4 kDa with Strep-tag) on SDS-PAGE gel (Fig. 3D). Smaller protein bands may be products from the autodegradation of DegP under reducing conditions^44^. We then examined EHEC single species biofilms by adding different concentrations of purified DegP for 24 h. Purified DegP repressed EHEC biofilm formation by up to 60-fold (5 µM) (Fig. 3E) but did not reduce the planktonic cell population (Fig. 3F). Furthermore, the addition of DegP during the dual species biofilm formation of EcN∆*degP*∆*kan* and EHEC resulted in decreased EHEC biofilms when the DegP concentration was 0.1 µM or higher (Fig. 3G). We observed that EcN∆*degP*∆*kan* biofilms were not affected by a low concentration of DegP (0.01 and 0.1 µM) but decreased with a high concentration of DegP (1 µM). This indicates that DegP secretion from the EcN wild-type is sufficiently balanced to inhibit EHEC biofilms but not affect the EcN biofilm itself. Corroborating the *degP* deletion and complementation results (Fig. 3B), these results suggest that DegP directly interacts with the EHEC cell surface, resulting in biofilm inhibition.

While DegP was found in the EcN supernatant but not in the BW or EHEC supernatants, the *degP* gene is present in BW and EHEC as well as EcN. DegP protein in BW is identical to that in EHEC, but DegP in EcN has three amino acid mismatches at positions 99, 301, and 392 compared to BW or EHEC DegP according to the amino acid sequence alignment (Supplementary Fig. 5). These amino acid mismatches of DegP in EcN may not affect the activity of DegP because only the mismatch at 99 appeared in the protease domain (1~259)^45,46^ and the active center (His105, Asp135 and Ser210)^46^ remains intact. Indeed, BW-DegP production in EcN inhibited EHEC biofilms by 10-fold (Supplementary Fig. 6) which is similar to the activity of EcN-DegP. To compare *degP* expression levels between different *E. coli* biofilms, we performed quantitative reverse transcription real-time PCR (qRT-PCR) of *degP* using total RNA isolated from single-species biofilms. We did not observe a distinguishable change in *degP* expression levels between EcN, BW, and EHEC by showing less than a 2-threshold value (*∆∆C*_*T*_) (Supplementary Table 5). In addition, the *degP* expression level of EcN biofilms from a dual-species setting was 7-fold higher than the expression level from single-species EcN biofilms (Supplementary Table 5), implying that DegP from EcN may be enriched in dual-species biofilms, resulting in EHEC biofilm repression. Together with the extracellular effect of purified DegP (Fig. 3E) and DegP presence in EcN supernatant (Supplementary Table 4), the qRT-PCR results indicate that the secretion of DegP from the periplasm of the cells to the extracellular space is critical to inhibit other biofilms.

### Effect of *degP*-deleted EcN on biofilm formation of *S. aureus* and *S. epidermidis*

**Figure 4:**
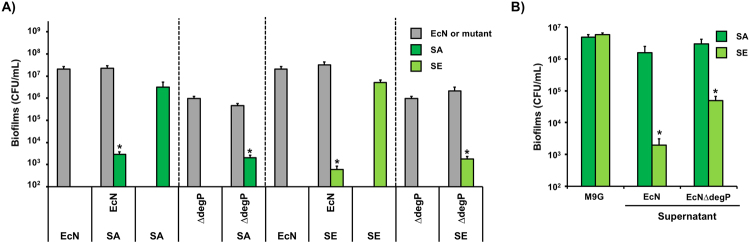
Effect of DegP on SA and SE biofilms. (**A**) Dual-species biofilms between EcN∆*degP* and SA or SE in M9G for 24 h at 37 °C without shaking. **(B)** SA and SE biofilm formation in supernatants from EcN wild-type and EcN∆*degP* at 37 °C for 24 h. Each data point is the average of at least two independent cultures with triplicates, and one standard deviation is shown. * represents a significant difference with a p-value < 0.01 by a two-tailed *t*-test.

EcN wild-type significantly inhibited the biofilm formation of *S. aureus* and *S. epidermidis* (Fig. 1B), the deletion of *degP* abolished the inhibition effect of EcN towards EHEC biofilms (Fig. 3B), and purified DegP repressed EHEC biofilms (Fig. 3E). To determine if the *degP* deletion of EcN influences the biofilm formation of *S. aureus* and *S. epidermidis*, we performed dual-species biofilm formation of these pathogens with EcN∆*degP*. SE biofilm formation with EcN∆*degP* was only slightly increased (3-fold) compared to the SE biofilms with the EcN wild-type (Fig. 4A), and SE biofilm formation in the EcN∆*degP* supernatant was 25-fold higher than that in the EcN supernatant (Fig. 4B). Thus, DegP may partially contribute to the activity of EcN in inhibiting SE biofilm formation. However, we observed a negligible effect of the *degP* deletion of EcN toward SA biofilm formation in the dual-species biofilm (Fig. 4A) and the SA biofilms in the EcN∆*degP* supernatant (Fig. 4B). Although the EcN wild type significantly inhibited SE and SA biofilm formation, the extracellular function of DegP was not clear in these pathogens. EcN may regulate SA and SE biofilms in ways other than controlling EHEC biofilms.

## Discussion

In this study, we showed that probiotic *E. coli* inhibits the biofilms of other *E. coli* strains as well as those of the pathogens *S. aureus* and *S. epidermidis* (Fig. 1B). The EcN supernatant showed a biofilm inhibition effect that was similar to that in dual-species biofilms (Fig. 2A), suggesting that the secreted EcN proteins are responsible for inhibiting the target biofilms. A proteomic analysis of *E. coli* supernatants and dual biofilm study using isogenic EcN mutants revealed that DegP contributes to the inhibition of EHEC biofilms (Fig. 3B). Although other *E. coli* strains produce DegP, the secretion of DegP, which is only available in EcN, is critical to repress other biofilms. Indeed, purified DegP repressed EHEC biofilm formation (Fig. 3E). Hence, we identified that probiotic *E. coli* outcompetes other biofilm formations via the extracellular function of DegP secreted from EcN.

DegP is a widely conserved periplasmic protein that shows chaperone functions at low temperature and has proteolytic activities at elevated temperatures^47^; DegP degrades misfolded and aggregated proteins from the inner-membrane and periplasmic space of *E. coli*^39^. Upon temperature elevation, proteolytic sites located in the central cavity of the DegP hexamer are accessible to bind substrates, but when DegP becomes a chaperone, the protease domain becomes inactive, thereby prohibiting substrate binding^45^. Such allosteric control of DegP has important effects in regulating proteolysis for protecting cells against various stresses^48,49^. DegP is closely related to the virulence of many pathogens, as the lack of DegP function increases the vulnerability of bacteria to stresses, resulting in a decreased secretion of virulence factors^46^. In this study, we found that extracellular DegP secreted by EcN represses EHEC biofilms (Fig. 3B and E), and the related mechanism may differ from the intracellular function of DegP, which protects the cells. Although extracellular DegP activity has not been widely studied, some pathogens, such as *Helicobacter pylori*^50^ and *Campylobacter jejuni*^51^, secrete DegP outside the cells and enhance their invasion to epithelial cells by cleaving E-cadherin, the cell adhesion protein located in the adherence junctions of epithelial cells^46^. Hence, although EcN is not a pathogen, EcN may have a DegP secretion system similar to the pathogens but uses DegP to outcompete other biofilms rather than invade epithelial cells. The purified DegP inhibited EHEC biofilms in single species (Fig. 3E) as well as in dual-species biofilms (Fig. 3G) but did not reduce the planktonic cell population (Fig. 3F). Thus, the extracellular function of DegP is not related to protease activity but instead likely modifies or binds the target cell surface, resulting in biofilm inhibition.

In addition to inhibiting pathogenic and commensal *E. coli* biofilms, EcN significantly outcompeted the Gram-positive pathogens *S. aureus* and *S. epidermidis* during biofilm formation (Fig. 1B). We found that EcN inhibits the growth rates of SA and SE (Supplementary Table 3), resulting in significant reductions of final cell density (Fig. 1C) along with biofilm repression. There may be some molecular regulation from EcN in these pathogens. DegP may partially contribute to inhibiting *S. epidermidis* biofilms but may not be effective for *S. aureus* biofilms (Fig. 4AB). Other proteins secreted by EcN may play important roles in inhibiting the cell growth and biofilm formation of *S. aureus* and *S. epidermidis*. Further studies revealing EcN-secreted proteins that control *S. aureus* and *S. epidermidis* biofilms are required. We did not observe population changes between *P. aeruginosa* and *E. coli* biofilms (EcN or BW) in dual-species biofilms (Fig. 1B). While *P. aeruginosa* promotes *E. coli* biofilms in nutrient-limited medium under sheared flow conditions^52^, the static biofilm formation environment in our study is different from the flow condition and may result in a reduced effect of *P. aeruginosa* in *E. coli* biofilms. We also did not observe any inhibitory effect of probiotic *E. coil* toward *P. aeruginosa* biofilms. *P. aeruginosa* has two quorum sensing systems mediated by acylated homoserine lactones (HSLs) (*i.e*., 3-oxo-C12-HSL and C4-HSL) that regulate many gene expressions related to biofilm formation and virulence factors^53,54^. If DegP or other secreted proteins by EcN are not related to regulating quorum sensing signaling, *P. aeruginosa* biofilms may not be affected by the probiotic *E. coli*. The results of selective biofilm inhibition by EcN in the dual-species biofilm formation with different pathogens show that the probiotic effect of EcN for controlling biofilm formation is strain-specific.

We demonstrated that probiotic *E. coli* outcompetes pathogenic *E. coli* during biofilm formation via a DegP-mediated interaction and significantly reduces the biofilm formation of *S. aureus* and *S. epidermidis*. Such biofilm inhibition is a unique characteristic of probiotic *E. coli*, as commensal *E. coli* does not exhibit this inhibition. Probiotic *E. coli* may use different mechanisms to control the biofilm formation of other bacteria due to the complexity of biofilm regulation, which should be explored further. This study revealed the influence of probiotic *E. coli* towards the biofilm formation of pathogens. The opposite side of the interaction, *i.e*., how pathogens affect the physiology and biofilm growth of probiotics, is equally interesting in understanding the molecular interactions in mixed bacterial communities. Particularly, elucidating the interaction mechanisms between probiotics and pathogens will provide insights for combating persistent biofilm-associated bacterial infections.

## Methods

### Bacterial strains and growth conditions

All experiments were conducted at 37 °C. The bacterial strains and plasmids used in this study are listed in Table 1.

**Table 1:** Strains and plasmids used in this study.

Strains and plasmids	Genotype/relevant characteristics	Source or reference
**Strains**		
*Escherichia coli* Nissle 1917 (EcN)	EcN wild-type strain	^61^
EcN str^R^	EcN with streptomycin resistance	^62^
EcN∆m	EcN Δ*mchBCDEF* Δ*mcmIA*, mutant lacking microcins M and H47	This study
EcN∆*degP*	EcN lacking *degP*	This study
EcN∆*degP*∆*kan*	EcN str^R^ lacking *degP* and *kan*^*R*^	This study
EcN∆*hslU*	EcN lacking *hslU*	This study
EcN∆*sat*	EcN lacking *sat*	This study
*E. coli* O157:H7	Enterohemorrhagic *E. coli* EDL933 strain, ATCC 43895	ATCC
*E. coli* BW25113	*lacI*^q^ *rrnB*_T14_ Δ*lacZ*_WJ16_ *hsdR*514 Δ*araBAD*_AH33_ Δ*rhaBAD*_LD78_	^33^
*E. coli* BW25113 *gadA* kan^R^	BW25113 with kanamycin resistance, Δ*gadA*Ωkan^R^	^33^
*Pseudomonas aeruginosa* PAO1	PAO1 wild-type strain	^34^
*Staphylococcus aureus* JE2	JE2 wild-type strain	^35^
*S. epidermidis* RP62A	RP62A wild-type strain	^36^
**Plasmids**		
pJL1-sfGFP	Kan^R^, *T7*::*sfGFP*, C-terminal Strep-tag, pY71-sfGFP	^63^
pDsRed-express	Amp^R^, *lac*::*DsRed-Express*	^64^
pKD4	Amp^R^, Kan^R^ cassette	^37^
pKD46	Amp^R^, *araBAD*, Red recombinase	^37^
pCP20	Amp^R^, Cm^R^, temperature-sensitive replication and thermal induction of Flp recombinase	^65^
pCA24N	Cm^R^; *lacI*^q^	^43^
pCA24N-*degP*	Cm^R^, *lacI*^q^::EcN-*degP*	This study
pCA24N-BW-*degP*	Cm^R^, *lacI*^q^::BW-*degP*	This study
pJL1-*degP*	Kan^R^, *T7*::*degP*, C-terminal Strep-tag	This study

str^R^, kan^R^, amp^R^, and cm^R^ indicate streptomycin, kanamycin, ampicillin, and chloramphenicol resistance, respectively.

### Quantification of dual-species biofilms and planktonic cells

An equal number of cells (approximately 4 × 10^6^ CFU/mL of each strain) from two bacterial strains grown overnight were inoculated into 1 mL M9G in a polypropylene culture tube (Falcon PN352006, Corning, Cambridge, MA, USA) without adding any antibiotics. After incubation at 37 °C for 24 h without shaking, the planktonic cell solutions were transferred to other containers and the culture tubes containing biofilms on the wall were rinsed three times with 1 mL PBS. Biofilm cells were carefully scraped from the wall of the culture tube with a pipette tip and re-suspended in 1 mL PBS via vortexing for 30 s. The antibiotic resistances of each bacterial strain were confirmed by plating the overnight cultures on the LB agar plates containing different antibiotics prior to the dual-species experiment. Only the cells expressing the corresponding antibiotic resistance gene grew during overnight incubation and were regarded as one population of dual-species biofilms. The number of planktonic and biofilm populations of each strain in a dual-species setting was determined by plating onto plain LB agar plates or LB agar plates supplemented with the appropriate antibiotic (Supplementary Table 1) after serial dilutions of the cell suspension^55^. We confirmed that scraping and vortexing removed nearly all biofilm cells from the surface of the culture tube (Supplementary Fig. 7) by staining remaining biofilm cells with 0.1% crystal violet^56^.

### Preparation of cell-free supernatant

Overnight cultures of EcN, BW, and EHEC were re-inoculated into fresh M9G to an initial cell concentration of 4 × 10^6^ CFU/mL and incubated for 16 to 18 h at 220 rpm. The supernatants were separated from the cells by filtration using a 0.22 µm pore size syringe filter (PN431229, Corning, Cambridge, MA, USA). Cell-free supernatants were used as growth media for EHEC biofilm formation and samples for mass spectrometry analysis. The supernatants were also separated with different molecular weight cut-off (MWCO) using a protein concentrator (Spin-X UF, Corning, Cambridge, MA, USA) via centrifugation at 14,000 × g until 90% of the initial volume was filtered. The supernatant SN-L (MW < 10 kDa) was collected as a flow-through using a 10 K MWCO concentrator. The concentrated and un-filtrated supernatant on the 10 K MWCO concentrator was diluted with M9G to restore their volume to be the same as the initial volume, and supernatant SN-M (10 kDa < MW < 30 kDa) was collected as a flow-through using a 30 K MWCO concentrator. The un-filtrated supernatant on the 30 K MWCO concentrator was then diluted with M9G to restore their volume to the initial volume.

### SDS-PAGE

Each single colony of EcN, BW, and EHEC strains was inoculated in fresh LB medium and incubated at 220 rpm at 37 °C. Overnight cultures were diluted 100-fold with M9G medium, incubated at 37 °C for 16 h, and then centrifuged at 14,000 × g for 5 min to remove the cell pellet. The supernatants were filter-sterilized using 0.2 µm pore size filters to eliminate any remaining cells. The sterilized supernatants were concentrated 100-fold through the 5 kDa MWCO protein concentrator (Spin-X UF, Corning, Cambridge, MA, USA) via centrifugation. The supernatant samples were denatured by incubating at 70 °C for 10 min and then loaded in the 4–12% SDS-PAGE gel (NuPAGE, Invitrogen, Carlsbad, CA, USA) in 1× NuPAGE MES SDS Running Buffer (Invitrogen, Carlsbad, CA, USA) for 35 min at 200 V to separate the protein bands. SeeBlue™ Plus2 Pre-stained Protein Standard (Invitrogen, Carlsbad, CA, USA) was used as a protein ladder for SDS-PAGE. Full-length protein gel for Figs 2B and 3D are shown in shown in Supplementary Fig. 8A and B.

### Mass Spectrometry

#### Protein preparation and digestion

The EcN, BW, and EHEC supernatants were prepared by removing cells through syringe filtration and concentrated 10-fold using a 5 k MWCO protein concentrator (Spin-X UF, Corning, Cambridge, MA) via centrifugation. The protein samples were diluted in a denaturing buffer of 50 mM Tris, pH 8 containing 8 M urea and reduced by adding 500 mM dithiothreitol (DTT) solution to a final concentration of 20 mM (1:25 dilution) at 60 °C for 30 min. The reduced protein sample was mixed with 1 M iodoacetamide solution to a final concentration of 40 mM (1:25 dilution) and incubated at room temperature for 30 min while protected from light. The alkylation reaction was quenched by adding 500 mM DTT solution to a final concentration of 10 mM (1:50 dilution). For protein digestion, trypsin solution was added to the sample to a final protease to protein ratio of 1:20 to 1:100 (w/w). The samples were incubated at 37 °C overnight and stored at −20 °C to stop digestion reactions. After overnight digestion, the peptides were eluted twice with 150 μL 0.1% formic acid (FA). The concentration of proteins and peptides collected in each step was measured using a NanoDrop 1000 (Thermo Scientific, San Jose, CA, USA). The digested peptides were then aliquoted, dried, and stored at −80 °C until further use or re-dissolved in 0.1% FA for LC−MS/MS analysis.

#### LC-MS/MS analysis

Fractions were run on a Thermo Fisher Orbitrap Velos Pro coupled with an Agilent NanoLC system (Agilent, Santa Clara, CA, USA) over a 60-min gradient. The LC columns (15 cm × 75 μm ID, Zorbax 300SB-C18) were purchased from Agilent. The samples were analyzed with a 60-minute linear gradient (0–35% acetonitrile with 0.1% formic acid), and data were acquired in a data-dependent manner, in which MS/MS fragmentation is performed on the top 10 most intense peaks of every full MS scan. RAW files were converted into.mgf files using MSConvert (from ProteoWizard)^57^.

#### Database searching

All MS/MS samples were analyzed using Mascot (Matrix Science, London, UK). Mascot was set up to search the SwissProt_2017_02 database (selected for *E. coli*, 23014 entries). Mascot was searched with a fragment ion mass tolerance of 0.50 Da and a parent ion tolerance of 10.0 ppm. Carbamidomethyl of cysteine was specified in Mascot as a fixed modification. The deamidation of asparagine and glutamine and the oxidation of methionine were specified in Mascot as variable modifications.

#### Criteria for protein identification

Scaffold (version Scaffold_4.7.5, Proteome Software Inc., Portland, OR, USA) was used to validate MS/MS-based peptide and protein identifications. Peptide identifications were accepted if they could be established at greater than 98.0% probability by the Scaffold Local False Discovery Rate algorithm. Protein identifications were accepted if they could be established at greater than 98.0% probability to achieve a false discovery rate of less than 1.0% and contained at least 1 identified peptide. Protein probabilities were assigned by the Protein Prophet algorithm^58^. Proteins that contained similar peptides and could not be differentiated based on MS/MS analysis alone were grouped to satisfy the principles of parsimony. Proteins sharing significant peptide evidence were grouped into clusters.

### Construction of EcN mutants

An EcN mutant strain lacking the microcin M and H47 genes (EcN∆m) was constructed using the λ Red recombinase system^37^ with H1P1 and H2P2 primers (Supplementary Table 6) that replace target EcN genome regions (*mchBCDEF* and *mcmIA*) with a kanamycin resistance gene cassette from pKD4 (Supplementary Fig. 3; Table 1). The deletion of the target genes and insertion of the kanamycin resistance cassette were confirmed by PCR using mic-F and mic-R primers (Supplementary Table 6). Similarly, EcN mutant strains lacking *degP*, *hslU*, or *sat* were constructed using corresponding primers (Supplementary Table 7), and the target genes in the EcN genome were replaced with the kanamycin resistance gene cassette. The deletion of the genes and insertion of the kanamycin resistance cassette were confirmed by PCR (Supplementary Table 7). For *degP*-deleted EcN, the kanamycin resistance cassette was also removed by Flp-FRT recombination using pCP20 plasmid (Table 1).

### Construction of plasmid pCA24N-*degP*

*degP* from EcN or BW was cloned into pCA24N plasmid (Table 1). The *degP* gene was amplified by PCR with degPfd and degPrv primers (Supplementary Table 7) using Pfu polymerase^59^ at 98 °C for 30 s, with 35 cycles of 98 °C for 30 s, 54 °C for 30 s, and 72 °C for 1 min, as well as a final extension of 72 °C for 5 min followed by DNA purification using a CyclePure kit (Omega Bio-Tek, Norcross, GA). The pCA24N plasmid and *degP* PCR products were digested with restriction enzymes BseRI and HindIII (New England Biolabs, Ipswich, MA, USA) and purified. The insert and vector were ligated by incubating with T4 DNA ligase (New England Biolabs, Ipswich, MA, USA) at 16 °C overnight. EcN cells were transformed with the ligated plasmid by electroporation (Bio-Rad, Hercules, CA, USA). The constructed plasmid pCA24N-*degP* was confirmed by sequencing with primers seqfd and seqrv (Supplementary Table 7).

### Confocal laser scanning microscopy and COMSTAT analysis

EcN and BW were transformed with plasmid pJL1-sfGFP, and EHEC was transformed with plasmid pDsRed-Express. Overnight cultures were diluted to a turbidity at 600 nm of 0.01 in 1 mL of M9G medium in the 24-well polystyrene microplates (Falcon PN351147, Corning, New York, NY) and incubated for 24 h at 37 °C without shaking. The plates were washed three times with 1 mL PBS before biofilm observation. The biofilms were observed using a LSM PASCAL laser Module (Carl Zeiss AG, Oberkochen, Germany) equipped in an Axiovert 200 M inverted microscope (Carl Zeiss AG, Oberkochen, Germany) with a 20X objective lens. Iilms were obtained using lasers with emission at 520 nm and excitation at 488 nm in one photomultiplier tube for green color and emission at 586 nm and excitation at 543 nm in the other photomultiplier tube for red color. At least six different positions from two independent biofilms were observed, and representative images were processed using IMARIS software (Bitplane, Belfast, UK). Color confocal images were converted to grey scale, the threshold was fixed to 30, and the biomass, surface coverage, mean thickness, and roughness coefficient (Supplementary Table 2) were determined using COMSTAT 2 image processing software^60^.

### Specific growth rate measurement in single- and dual-species cultures

Overnight LB cultures were diluted in fresh M9G medium to the initial cell concentration of 4 × 10^6^ CFU/mL. 2.25 mL of diluted culture was added into each well of a 6-well culture plate (PN3450, Corning, Cambridge, MA, USA) and incubated at 37 °C for 12 h without shaking. Cell growth (OD_600_) was measured every hour to calculate the specific growth rate. For dual-species culture, a transwell permeable support membrane (0.4 µm pore size) was added to the well and the same initial concentration of EcN was added on top of the well so that the target cells in the bottom of the well and the EcN cells at the top of the well were separated but so that the nutrients, proteins, and metabolites were exchanged through the permeable membrane. The growth of the target cells was measured every hour by removing the transwell permeable membrane.

### Quantitative reverse transcription real-time PCR

#### Biofilm cell pellet preparation

Overnight cultures of EcN, BW, and EHEC were diluted to be OD_600_ of 0.05 in 2 mL of fresh M9G in test tubes (PN352006, Corning, Cambridge, MA, USA). After 8 h incubation at 37 °C without shaking, planktonic cells were discarded, and the tubes were washed twice with 2 mL PBS. The biofilm cells that formed on the walls of the tube were collected by scraping and resuspending with 1 mL PBS. The biofilm cell pellets were obtained by centrifugation in a manner similar to the methods of the EcN biofilm cell isolation above. For biofilm cell pellet isolation from the dual-species biofilm, we used a transwell plate as described in the specific growth rate measurement setting. After 8 h incubation of the EcN (bottom well) and EHEC (top well) dual biofilms, the EcN biofilms that formed on the bottom wall were collected by washing, scraping, and resuspending. The EcN biofilm cell pellet was obtained by centrifugation.

#### Total RNA isolation and qRT-PCR

Total RNA was isolated from the biofilm cell pellets using a ZR Fungal/Bacterial RNA MiniPrep™ kit (Zymo Research, Orange, CA, USA) and quantified by a UV 1600-PC spectrophotometer (VWR, Darmstadt, Germany) reading OD at 260 nm and 280 nm. 50 ng of total RNA was mixed with an iTaq^TM^ Universal SYBR green one-step kit (Bio-Rad, Hercules, CA, USA) by following the manufacturer’s protocol, and qRT-PCR was performed using *degP* and *rrsG* RT-PCR primers (Supplementary Table 7) in a CFX Connect Real-Time PCR Detection System (Bio-Rad, Hercules, CA, USA). The expression levels of *degP* and *rrsG*, a housekeeping gene of 16 S ribosomal RNA of *rrnG* operon, were monitored, and the *C*_*T*_ value was compared to analyze the *degP* expression levels in different samples.

### DegP purification

The *degP* gene was amplified from EcN using DegP-F and DegP-R primers (Supplementary Table 7), which add Strep-tag at the C-terminus and NdeI and SalI restriction enzyme sites at 5′ and 3′ ends, respectively. The PCR fragment was double-digested by NdeI and SalI (New England Biolabs, Ipswich, MA, USA) and ligated into pJL1 vector at the same restriction sites. The ligation mix was electroporated into *E. coli* BL21(DE3) Star competent cells. To produce DegP protein, an overnight culture of BL21(DE3) Star harboring pJL1-*degP* was diluted 100-fold in 250 mL of LB with 50 μg/mL of kanamycin in a 1-L flask and incubated at 37 °C at 220 rpm until OD_600nm_ ~ 0.5. Then, 1 mM of isopropyl β-D-1-thiogalactopyranoside was added to induce DegP production and incubated at 18 °C at 220 rpm overnight. Cells were harvested at 5,000 rpm at 4 °C for 15 minutes and resuspended with 1 mg/mL lysis buffer (50 mM NaH_2_PO_4_, 300 mM NaCl, 10 mM Imidazole, adjusted pH to 8.0 with NaOH). The cells were lysed by sonication (Qsonica, Newtown, CT), and cell debris was removed by centrifugation at 12,000 rpm at 4 °C for 10 minutes. DegP was purified using a Strep-Tactin column following the manufacturer’s suggested protocol (IBA, Göttingen, Germany) and concentrated by Spin-X^®^ UF ultrafiltration concentrators (Corning Incorporated, Corning, NY, USA). Five hundred microliters of elution were added into the 30 k MWCO concentrator and spun down at 12,000 rpm at 4 °C for 10 minutes. The size of DegP was confirmed by loading on SDS-PAGE. The DegP concentration was 7.2 mg/mL, as measured by a Quick-Start Bradford protein assay kit (Bio-Rad, Hercules, CA, USA).

### EHEC biofilm assay with DegP addition

The overnight culture of EHEC was diluted in 1 mL of M9G in a polypropylene culture tube (Falcon PN352006, Corning, Cambridge, MA) with 5 µM, 2 µM, 1 µM, 0.1 µM, and 0.01 µM of DegP. After incubation at 37 °C for 24 h without shaking, the biofilm CFU was quantified as described above.

### Statistical analysis

Statistical significance was assessed using two-tailed *t*-tests between the target strain’s population in the dual-species biofilms and its single-species biofilm population. The null hypothesis stated that the means of two samples are equal. Statistical significance was accepted for p values < 0.01 and indicated by asterisks in figures. All reported data are the average ± standard deviation from at least two experiments with triplicates.

## Electronic supplementary material

Supplemantary Information
