# Supplementary material for: Probiotic Escherichia coli inhibits biofilm formation of pathogenic E. coli via extracellular activity of DegP
Source: Sci Rep. 2018 Mar 21;8:4939. doi: 10.1038/s41598-018-23180-1 (PMC5862908; doi:10.1038/s41598-018-23180-1)
Supplement: Supplementary file 1 — Supplemantary Information [file 41598_2018_23180_MOESM1_ESM.pdf]

Supplementary Information

Probiotic *Escherichia coli* inhibits biofilm formation of pathogens via extracellular activity of DegP

Kuili Fang, Xing Jin and Seok Hoon Hong*

Department of Chemical and Biological Engineering, Illinois Institute of Technology,

Chicago, IL 60616, USA

*Corresponding author: shong26@iit.edu

Tel: (+1) 312 567 8950

Supplementary Table 1. Antibiotics used for dual-species biofilm quantification on LB agar plates. '0' indicates no antibiotics used. str, kan, amp, and cipro indicate streptomycin (100 μg/mL), kanamycin (50 μg/mL), ampicillin (100 μg/mL), and ciprofloxacin (5 μg/mL), respectively. EcN wild-type or streptomycin-resistant strains and BW wild-type or kanamycin-resistant strains were used depending on the antibiotic resistance of the other strain in dual-species biofilms. EcN, BW, EHEC, PA, SA, and SE indicate probiotics *E. coli* Nissle 1917, *E. coli* BW25113, enterohemorrhagic *E. coli* EDL933, *P. aeruginosa* PAO1, *S. aureus* JE2, and *S. epidermidis* RP62A, respectively.

**Antibiotics for quantifying biofilm population between EcN and the other strain**				**Antibiotics for quantifying biofilm population between BW and the other strain**			
EcN	0/str	BW	kan				
EcN	0/str	EHEC	0	BW	kan	EHEC	0
EcN	str	PA	amp	BW	kan	PA	amp
EcN	str	SA	cipro	BW	0	SA	cipro
EcN	0	SE	str	BW	0	SE	str

Supplementary Table 2. COMSTAT analysis of biofilms observed by confocal microscope corresponding to **Fig. 1D**.

**Biofilms**		**Biomass, μm^3/μm^2**	**Substratum coverage, %**	**Mean thickness, μm**	**Roughness coefficient**
EcN	EcN (green)	9 ± 1	60 ± 20	9 ± 1	0.5 ± 0.2
EcN + EHEC	EcN (green)	8 ± 3	5 ± 2	4 ± 2	1.6 ± 0.2
	EHEC (red)	5 ± 2	24 ± 2	7 ± 3	1.5 ± 0.3
EHEC	EHEC (red)	60 ± 10	90 ± 10	60 ± 10	1.5 ± 0.5
BW + EHEC	BW (green)	25 ± 4	13 ± 4	35 ± 3	1.15 ± 0.02
	EHEC (red)	46 ± 9	70 ± 10	50 ± 10	1.16 ± 0.04
BW	BW (green)	27 ± 5	60 ± 20	23 ± 2	0.18 ± 0.07

Supplementary Table 3. Specific growth rates for each strain in the single culture and dual culture with EcN using transwell plates.

**Strain Name**	**Specific growth rate in single culture (1/h)**	**Specific growth rate in dual culture with EcN (1/h)**
EcN	0.44±0.04	-
BW	0.335±0.006	0.19±0.07
EHEC	0.426±0.005	0.43±0.01
PAO1	0.36±0.05	0.37±0.01
SA	0.27±0.01	0.09±0.01
SE	0.30±0.07	0.08±0.02

Supplementary Table 4. List of proteins uniquely identified in EcN supernatant from mass spectrometry analysis.

Protein Name	Accession Number	Molecular Weight	Characters
**Protease**			
ClpP	CLPP_ECO24	23 kDa	ATP-dependent Clp protease proteolytic subunit
DegP	DEGP_ECO57	49 kDa	Periplasmic serine endoprotease DegP
HslU	HSLU_ECO24	50 kDa	ATP-dependent protease ATPase subunit HslU
Sat	SAT_ECOL6	140 kDa	Serine protease sat autotransporter
**Metabolism**			
AceE	ODP1_ECO57	100 kDa	Pyruvate dehydrogenase E1 component
AhpF	AHPF_ECOLI	56 kDa	Alkyl hydroperoxide reductase subunit F
ArgG	ASSY_ECO27	50 kDa	Argininosuccinate synthase
CysI	CYSI_ECO7I	64 kDa	Sulfite reductase [NADPH] hemoprotein beta-component
BabD	GABD_ECOLI	52 kDa	Succinate-semialdehyde dehydrogenase [NADP(+)] GabD
IlvC	ILVC_ECO24	54 kDa	Cluster of Ketol-acid reductoisomerase [NADP(+)]
IucD	IUCD_ECOLX	49 kDa	L-lysine N6-monooxygenase
YehH	MOLR_ECOLI	141 kDa	Putative molybdate metabolism regulator
SucD	SUCD_ECO57	30 kDa	Succinate--CoA ligase [ADP-forming] subunit alpha
SerA	SERA_ECO57	44 kDa	D-3-phosphoglycerate dehydrogenase
LysA	DCDA_ECOLI	46 kDa	Diaminopimelate decarboxylase
Pta	PTA_ECOLI	77 kDa	Phosphate acetyltransferase
UxaC	UXAC_ECO24	54 kDa	Uronate isomerase
MelA	AGAL_ECOLI	51 kDa	Alpha-galactosidase
**Chaperone related**			
FocC	FOCC_ECOL6	25 kDa	Chaperone protein FocC
GroL1	CH601_ECOK1	57 kDa	60 kDa chaperonin 1
GrpE	GRPE_ECO24	22 kDa	Protein GrpE
**Flagella**			
FlgF	FLGF_ECOLI	26 kDa	Flagellar basal-body rod protein FlgF
FlgH	FLGH_ECO24	25 kDa	Flagellar L-ring protein
FlgK	FLGK_ECOLI	58 kDa	Flagellar hook-associated protein 1
FlgL	FLGL_ECOLI	34 kDa	Flagellar hook-associated protein 3
FlgM	FLGM_ECOLI	10 kDa	Negative regulator of flagellin synthesis
FliC	FLIC_ECOLI	51 kDa	Flagellin
FliD	FLID_ECOLI	48 kDa	Flagellar hook-associated protein 2
FliE	FLIE_ECO27	11 kDa	Flagellar hook-basal body complex protein FliE
**Stress-related**			
BhsA	BHSA_ECO57	9 kDa	Multiple stress resistance protein BhsA

DctA	DCTA_ECO57	45 kDa	Aerobic C4-dicarboxylate transport protein
EntF	ENTF_ECO57	142 kDa	Enterobactin synthase component F
PspA	PSPA_ECO57	25 kDa	Phage shock protein A
**Transcription/translation**			
HisG	HIS1_ECO24	33 kDa	ATP phosphoribosyltransferase
HisS	SYH_ECO24	47 kDa	Histidine--tRNA ligase
IscR	ISCR_ECO24	17 kDa	HTH-type transcriptional regulator IscR
IutA	IUTA_ECOLX	81 kDa	Ferric aerobactin receptor
PheT	SYFB_ECO57	87 kDa	Phenylalanine--tRNA ligase beta subunit
PhoH	PHOH_ECO57	39 kDa	Protein PhoH
ProS	SYP_ECO24	64 kDa	Proline--tRNA ligase
PurL	PUR4_ECO57	141 kDa	Phosphoribosylformylglycinamidine synthase
PurM	PUR5_ECO27	37 kDa	Phosphoribosylformylglycinamidine cyclo-ligase
RcsB	RCSB_ECO57	24 kDa	Transcriptional regulatory protein RcsB
Rho	RHO_ECO57	47 kDa	Transcription termination factor Rho
RpoA	RPOA_ECO24	37 kDa	DNA-directed RNA polymerase subunit alpha
RpoB	RPOB_ECO24	151 kDa	DNA-directed RNA polymerase subunit beta
RpsC	RS3_ECO24	26 kDa	30S ribosomal protein S3
SlyA	SLYA_ECO24	16 kDa	Transcriptional regulator SlyA
TauA	TAUA_ECOLI	34 kDa	Taurine-binding periplasmic protein
ThiB	THIB_ECOLI	36 kDa	Thiamine-binding periplasmic protein
TrpB	TRPB_ECO24	43 kDa	Cluster of Tryptophan synthase beta chain
**Uncharacterized**			
YcfJ	YCFJ_ECOL6	19 kDa	Uncharacterized protein YcfJ
YjeI	YJEI_ECOL6	12 kDa	Uncharacterized protein YjeI

Supplementary Table 5. qRT-PCR of *degP* in *E. coli* single species biofilms and dual species biofilms in M9G medium at 37°C for 8 h. C_T is the threshold cycle of the target genes. ΔC_T was calculated by subtracting the C_T of *rrsG* (housekeeping gene) from the C_T of *degP*. $\Delta\Delta C_T$ was calculated by subtracting the ΔC_T of EcN from the ΔC_T of BW or EHEC, or EcN in dual-species biofilms. Fold change was calculated by $2^{-\Delta\Delta CT}$. Standard deviation is shown for ΔC_T and $\Delta\Delta C_T$ (n=4).

**Gene**	**qRT-PCR**		
	ΔC_T	$\Delta\Delta C_T$	**Fold change**
***degP* expression in single-species biofilms in test tubes**			
EcN	11.9±0.4		
BW	10.3±0.6	-1.7±0.7	3.2
EHEC	12.7±0.4	0.8±0.5	0.6
***degP* expression of EcN in dual-species biofilms in transwell plates**			
EcN	12.7±0.5		
EcN in dual	9.9±0.4	-2.9±0.6	7.3

Supplementary Table 6. Primers used for constructing and confirming EcNΔm. The uppercase character sequences of H1P1 and H2P2 overlap part of the 5'- and 3'-end of the kanamycin gene from pKD4, respectively.

**Name**	**Purpose**	**Primer sequence (5' → 3)**
H1P1	Upstream flanking of EcN microcin genes	gggattcgaaggattctggtctggtcaggctggaaaaacggaagttaaatatgatggagGTGTAGGCTGGAGCTGCTTC
H2P2	Downstream flanking of EcN microcin genes	cataggcaccattatcatataatgaagcacctgtaacaggtgcttcattaacaatataaggCATATGAATATCCTCCTTTAG
mic-f	Checking microcin gene deletion	gacggctatattcagtttacctattac
mic-r		ggcttctatcagttatactcattaac

Supplementary Table 7. Primers used for constructing and confirming EcNΔ*degP*, EcNΔ*sat*, EcNΔ*hslU*, pCA24N-*degP*, and pJL1-*degP*. Underlined italic text indicates restriction enzyme recognition sites: BseRI for degPfd, HindIII for degPrv, NdeI for degP-F, and SalI for degP-R. The uppercase character sequences of degP_Kan1, sat_Kan1, and hslU_Kan1 are part of the 5'- end of the kanamycin gene from pKD4, and the uppercase character sequences of degP_Kan2, sat_Kan2, and hslU_Kan2 are part of the 3'- end of the kanamycin gene from pKD4. The uppercase character sequences of degPfd, degPrv, degP-F, and degP-R are part of the 5' and 3'- end of the *degP* gene binding sites. The bold character sequence of degP-R indicates Strep-tag.

Name	Purpose	Primer sequence (5' → 3')
degP_up	Checking *degP* deletion	cgacctctatgcgtgggatg
degP_down		ggtatcaagatgccagccag
degP_Kan1	Upstream flanking of *degP*	ctataaaacgaatctgaagaacacagcaattttgcgttatctgttaatcgagactgaaatGTGTAGGCTGGAGCTGCTTC
degP_Kan2	Downstream flanking of *degP*	gaagttcacagattgtaaaaggagaaccccttcccgttttcaggaaggggttgagggaaaCATATGAATATCCTCCTTAG
sat_up	Checking *sat* deletion	gaatatgtgcattttttgc
sat_down		gcaaaaaaaaacaaaaaacg
sat_Kan1	Upstream flanking of *sat*	cacagggtaatacatattctctccggtagaagaagggccgcaaacgcggcccgggctgttGTGTAGGCTGGAGCTGCTTC
sat_Kan2	Downstream flanking of *sat*	gttactaataattttaattcttaatactttgttcattcaataaatgagttgagagaatatCATATGAATATCCTCCTTAG
hslU_up	Checking *hslU* deletion	gaccttgccaccatgccagc
hslU_down		gctccggcggcccttacgcc
hslU_Kan1	Upstream flanking of *hslU*	cgccaataaaaatggggcctttcagccccatcaaacaatgatgaaaatgattgaacgcgaGTGTAGGCTGGAGCTGCTTC
hslU_Kan2	Downstream flanking of *hslU*	catctataccaaccatttccacaccatcgaagaattaagctacaaagcgtaaggatctccCATATGAATATCCTCCTTAG
degPfd	Forward primer for *degP* cloning	attaaa*gaggagaaattaactA*TGAAAAAAACCACATTAGC
degPrv	Reverse primer for *degP* cloning	ctaatt*aagctt*caaaagaTTACTGCATTAACAGGTAGATG
seqfd	Checking *degP* insertion in pCA24N	gccctttcgtcttcacctcg
seqrv		gaacaaatccagatggagttctgaggtcatt
degP-F	Forward primer for *degP* cloning into pJL1 plasmid	attttgtttaactttaagaaggagatata*catATG*AAAAAAACCACATTAG
degP-R	Reverse primer for *degP* cloning into pJL1 plasmid	cgggctttgttagcagccg*gtcgac*tta**tttttcgaactgcggatggctcca**CTGCATTAACAGGTAG
rrsG_fwd	*rrsG* qRT-PCR forward and reverse primers	tattgcacaatgggcgcaag
rrsG_rev		acttaacaaaccgcctgcgt
degP_fwd	*degP* qRT-PCR forward and reverse primers	gagacttcttcagcaacgac
degP_rev		aacggttgtgctaccttcta

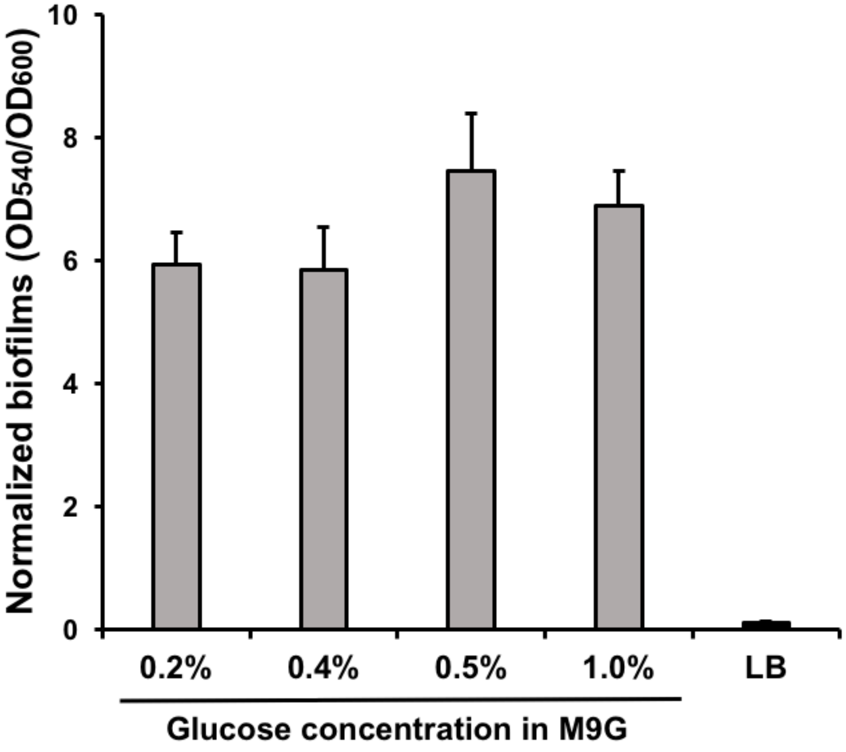

Supplementary Figure 1. Normalized EHEC biofilms in LB and M9G media with different glucose concentrations at 37°C for 24 h.

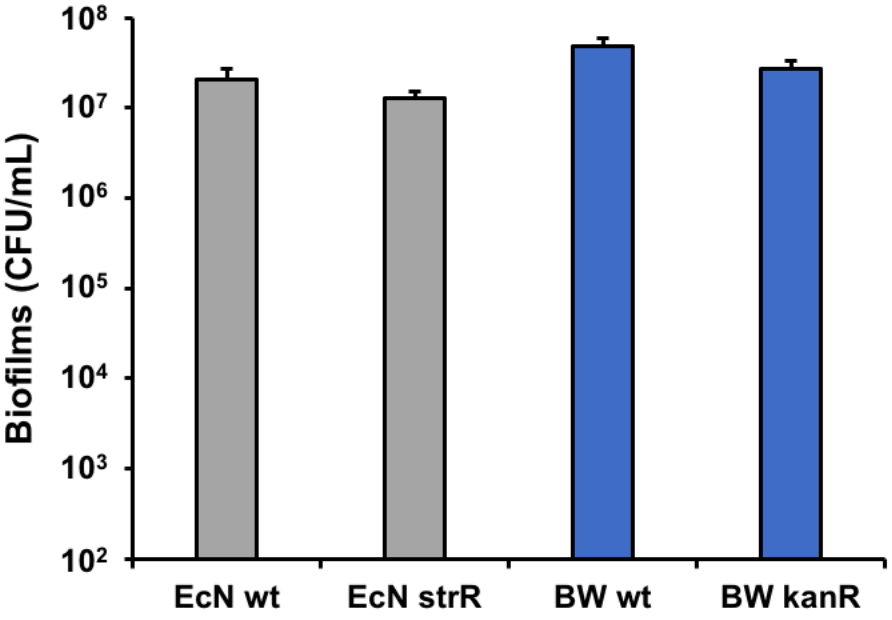

Supplementary Figure 2. Biofilm population comparisons between wild-type and antibiotic-resistant strains of probiotic *E. coli* (EcN) and BW25113 (BW). strR and kanR indicate streptomycin resistance and kanamycin resistance, respectively. Each data point is the average of at least six independent cultures, and one standard deviation is shown.

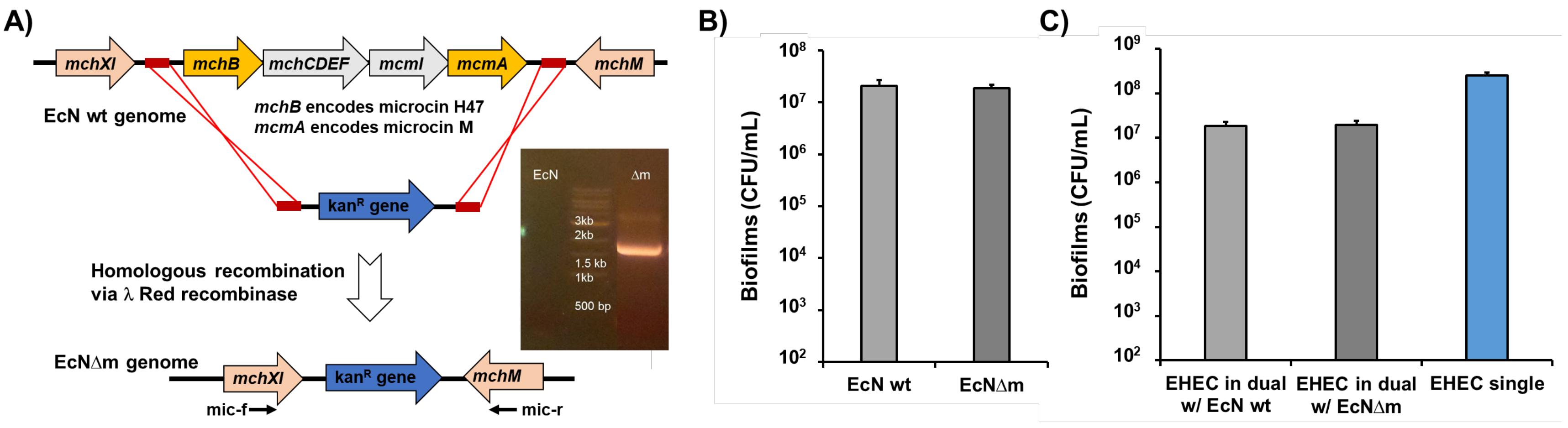

Supplementary Figure 3. Construction and biofilm test of EcN mutant lacking microcin H47 and M (EcNΔm). (**A**) Genes encoding microcin H47 and M were replaced by kanamycin resistance (kan^R) cassette by homologous recombination. The gene deletion was confirmed by PCR using mic-f and r primers (**Supplementary Table 6**). (**B**) Single-species biofilms of EcNΔm and the wild-type (wt) were formed for 24 h at 37°C in polypropylene test tubes. (**C**) EHEC biofilm population in the dual-species biofilms with EcNΔm or wt were compared. Each data point in (B) and (C) is the average of at least four independent cultures, and one standard deviation is shown.

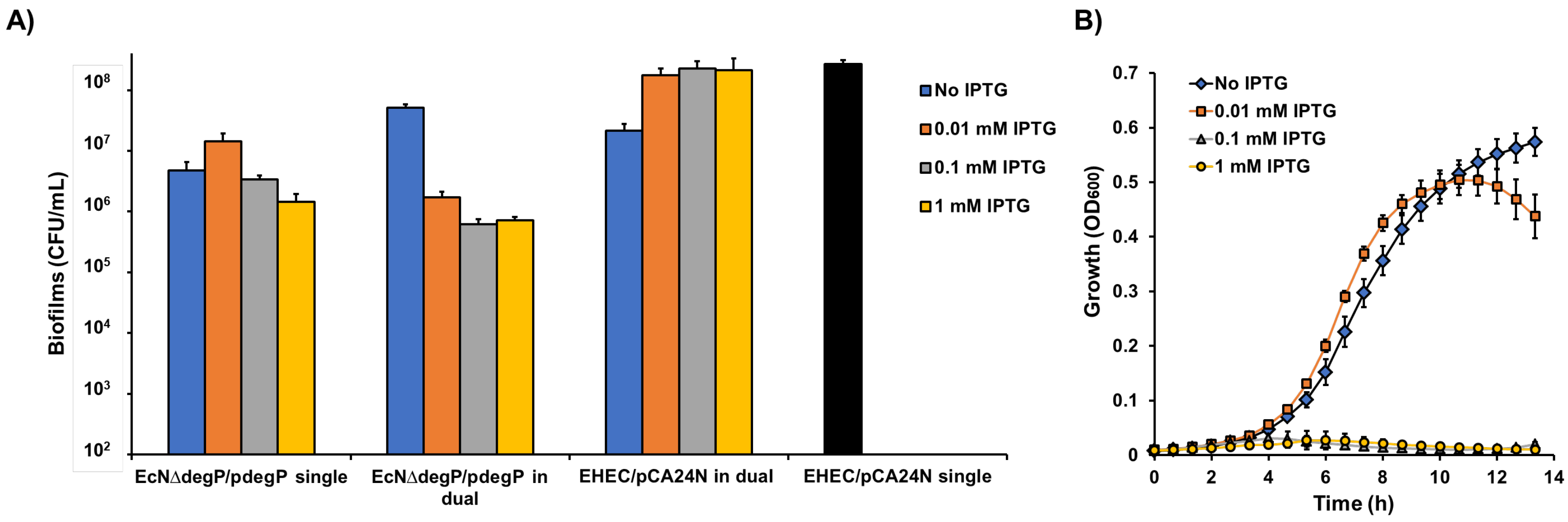

Supplementary Figure 4. DegP effects on EHEC biofilms and EcN cell growth. (**A**) Dual species biofilms between EcNΔ*degP*/pCA24N-*degP* and EHEC/pCA24N by overexpressing *degP* with the additions of 0.01 mM, 0.1 mM, and 1 mM of IPTG in M9G for 24 h at 37°C without shaking. (**B**) Growth of EcNΔ*degP*/pCA24N-*degP* with 0.01 mM, 0.1 mM, and 1 mM of IPTG. Each data point is the average of at least three independent cultures.

```
         1*************************************************50
EcN      MKKTTLALSALALSLGLALSPLSATAAETSSATTAQQMPSLAPMLEKVMP
BW       MKKTTLALSALALSLGLALSPLSATAAETSSATTAQQMPSLAPMLEKVMP
EHEC     MKKTTLALSALALSLGLALSPLSATAAETSSATTAQQMPSLAPMLEKVMP

        51********************************************** *100
EcN      SVVSINVEGSTTVNTPRMPRNFQQFFGDDSPFCQEGSPFQSSPFCQGGLG
BW       SVVSINVEGSTTVNTPRMPRNFQQFFGDDSPFCQEGSPFQSSPFCQGGQG
EHEC     SVVSINVEGSTTVNTPRMPRNFQQFFGDDSPFCQEGSPFQSSPFCQGGQG

       101*************************************************150
EcN      GNGGGQQQKFMALGSGVIIDADKGYVVTNNHVVDNATVIKVQLSDGRKFD
BW       GNGGGQQQKFMALGSGVIIDADKGYVVTNNHVVDNATVIKVQLSDGRKFD
EHEC     GNGGGQQQKFMALGSGVIIDADKGYVVTNNHVVDNATVIKVQLSDGRKFD

       151*************************************************200
EcN      AKMVGKDPRSDIALIQIQNPKNLTAIKMADSDALRVGDYTVAIGNPFGLG
BW       AKMVGKDPRSDIALIQIQNPKNLTAIKMADSDALRVGDYTVAIGNPFGLG
EHEC     AKMVGKDPRSDIALIQIQNPKNLTAIKMADSDALRVGDYTVAIGNPFGLG

       201*************************************************250
EcN      ETVTSGIVSALGRSGLNAENYENFIQTDAAINRGNSGGALVNLNGELIGI
BW       ETVTSGIVSALGRSGLNAENYENFIQTDAAINRGNSGGALVNLNGELIGI
EHEC     ETVTSGIVSALGRSGLNAENYENFIQTDAAINRGNSGGALVNLNGELIGI

       251*************************************************300
EcN      NTAILAPDGGNIGIGFAIPSNMVKNLTSQMVEYGQVKRGELGIMGTELNS
BW       NTAILAPDGGNIGIGFAIPSNMVKNLTSQMVEYGQVKRGELGIMGTELNS
EHEC     NTAILAPDGGNIGIGFAIPSNMVKNLTSQMVEYGQVKRGELGIMGTELNS

       301 ************************************************350
EcN      DLAKAMKVDAQRGAFVSQVLPNSSAAKAGIKAGDVITSLNGKPISSFAAL
BW       ELAKAMKVDAQRGAFVSQVLPNSSAAKAGIKAGDVITSLNGKPISSFAAL
EHEC     ELAKAMKVDAQRGAFVSQVLPNSSAAKAGIKAGDVITSLNGKPISSFAAL

       351****************************************** ********400
EcN      RAQVGTMPVGSKLTLGLLRDGKQVNVNLELQQSSQNQVDSSTIFNGIEGA
BW       RAQVGTMPVGSKLTLGLLRDGKQVNVNLELQQSSQNQVDSSSIFNGIEGA
EHEC     RAQVGTMPVGSKLTLGLLRDGKQVNVNLELQQSSQNQVDSSSIFNGIEGA

       401*************************************************450
EcN      EMSNKGKDQGVVVNNVKTGTPAAQIGLKKGDVIIGANQQAVKNIAELRKV
BW       EMSNKGKDQGVVVNNVKTGTPAAQIGLKKGDVIIGANQQAVKNIAELRKV
EHEC     EMSNKGKDQGVVVNNVKTGTPAAQIGLKKGDVIIGANQQAVKNIAELRKV

       451************************474
EcN      LDSKPSVLALNIQRGDSTIYLLMQ
BW       LDSKPSVLALNIQRGDSTIYLLMQ
EHEC     LDSKPSVLALNIQRGDSTIYLLMQ
```

Supplementary Figure 5. Alignment of DegP amino acid sequences from EcN, BW, and EHEC. Three amino acid mismatches of EcN DegP are shown with red font and yellow highlight.

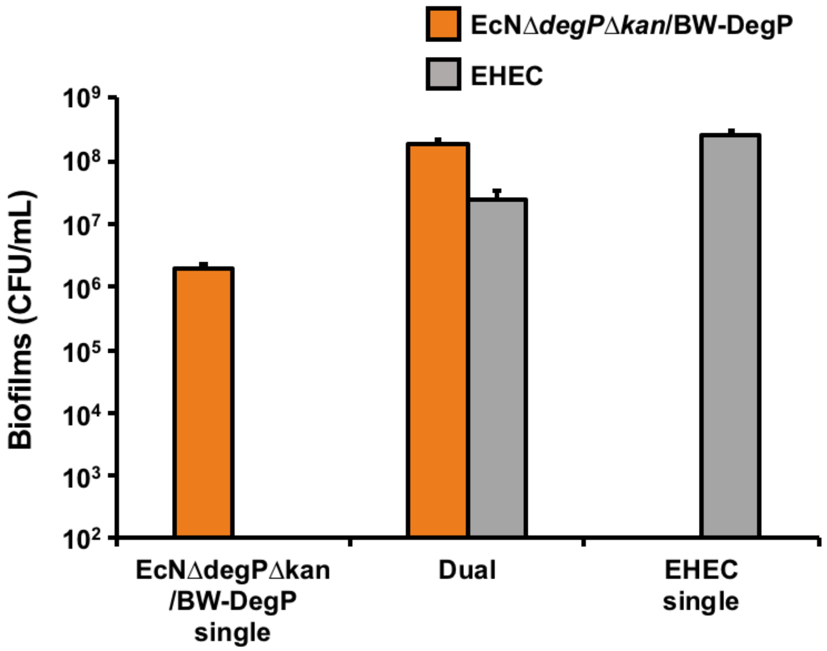

Supplementary Figure 6. Effect of BW-DegP on EHEC biofilm. Dual species biofilms between EcNΔ*degP*Δ*kan*/pCA24N-BW-*degP* and EHEC/pCA24N in M9G for 24 h at 37°C without shaking. Each data point is the average of at least three independent cultures.

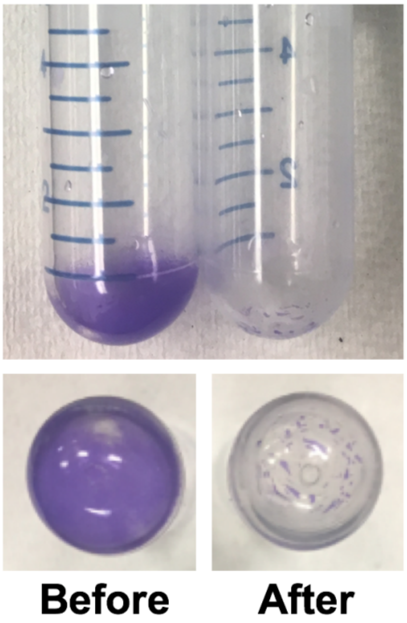

Supplementary Figure 7. Confirmation of remaining biofilm cells before and after the removal. Biofilm cells were removed by scraping and vortexing for population quantification. The remaining cells on the tube before and after the removal were visualized by staining with 0.1% crystal violet followed by 20 min incubation and washing with 1 mL PBS.

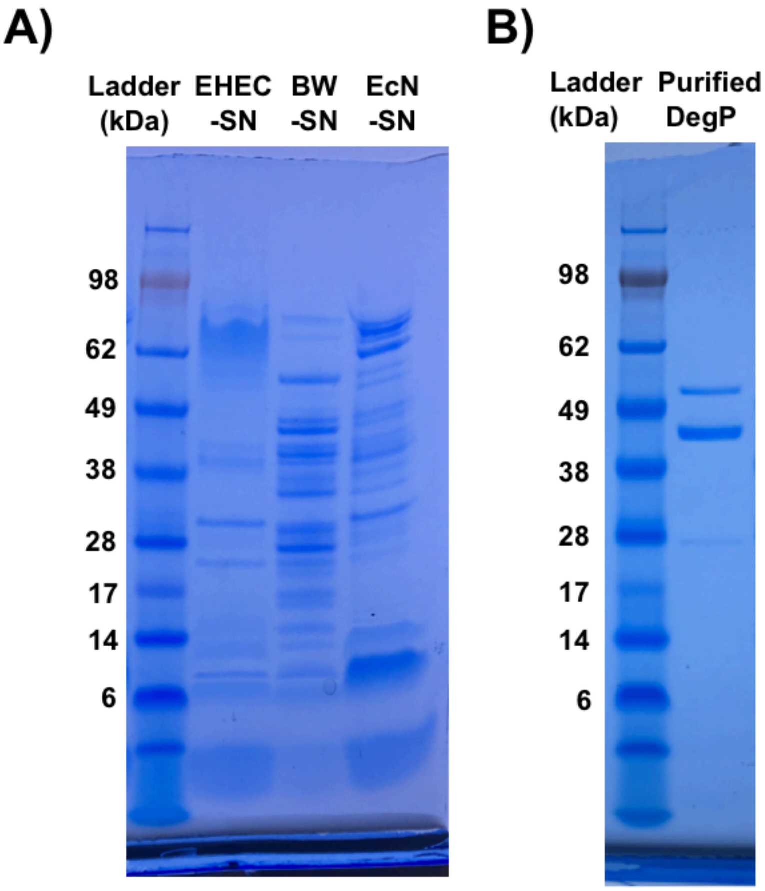

Supplementary Figure 8. (**A**) Full-length SDS-PAGE gel of EcN, BW, and EHEC supernatants of **Fig. 2B**. (**B**) Full-length SDS-PAGE gel of purified DegP of **Fig. 3D**. The samples were loaded in the 4-12% SDS-PAGE gel.
